# Identifying CEACAM1 as a potential prognostic biomarker for basal-like breast cancer by bioinformatics analysis and *in vitro* experiments

**DOI:** 10.7150/jca.101636

**Published:** 2024-10-21

**Authors:** Boke Zhang, Ran Liu, Haixia Huang, Chuanzhu Wang, Changcheng Yang

**Affiliations:** 1 Department of Clinical Laboratory Center, The First Affiliated Hospital of Anhui University of Chinese Medicine, Hefei, China.; 2 Department of Cancer center, The First Dongguan Affiliated Hospital, Guangdong Medical University, Dongguan, China.; 3 Department of Medical Oncology, The First Affiliated Hospital of Hainan Medical University, Haikou, China.; 4 Department of Clinical Laboratory, Anhui No.2 Provincial People's Hospital, Hefei, China.; 5 Department of Medical Oncology, People's Hospital of Wanning, Wanning, Hainan Province, China.

**Keywords:** Breast cancer, Basal-like breast cancer (BLBC), CEACAM1, Prognosis, Biomarker

## Abstract

**Background:** Carcinoembryonic antigen related cell adhesion molecule-1 (CEACAM1) is a very important intercellular adhesion molecule, and its prognostic relevance to breast cancer (BC), especially basal-like breast cancer (BLBC), remains poorly understood.

**Methods:** CEACAM1 mRNA expression data for BC were sourced from the Cancer Genome Atlas (TCGA) database. Kaplan-Meier survival analysis and Cox regression analysis were used to evaluate the prognostic relationship between CEACAM1 expression and BC. Signaling pathways associated with CEACAM1 were analysed using Gene Set Enrichment Analysis (GSEA) and Kyoto Encyclopedia of Genes and Genomes (KEGG) analysis. Moreover, cell counting kit-8 (CCK-8), flow cytometry, transwell and wound-healing assays were employed to identify the biological functions of CEACAM1 in BLBC.

**Results:** CEACAM1 was correlated with overall survival (OS) of BLBC patients. Compared with the subgroup with better prognosis, the levels of CEACAM1 mRNA expression were significantly lower in the subgroup of BLBC with poorer prognosis. Both univariate and multivariate Cox regression analysis suggested that down-regulation of CEACAM1 expression may be an independent factor for poor prognosis in BLBC patients. GSEA and KEGG analysis revealed that CEACAM1 was negatively related with signaling pathways including extracellular matrix (ECM) receptor interaction, focal adhesion, and cell adhesion. The results of *in vitro* experiments indicated that CEACAM1 not only induced apoptosis of BLBC cells, but also inhibited the invasive and metastatic ability of cancer cells.

**Conclusions:** CEACAM1 may contribute to improving the OS of BLBC patients due to its ability to inhibit the proliferation and metastasis of cancer cells. Therefore, CEACAM1 could be used as a potential prognostic biomarker and therapeutic target in BLBC.

## Introduction

As a common malignant tumor all over the world, breast cancer (BC) is one of the leading causes of death in women^1^. Despite the fact that much progress accomplished in the treatment of breast cancer in recent years, some patients still died from cancer recurrence, metastasis and drug resistance^2^, ^3^. Basal-like breast cancer (BLBC) is a specific category of invasive BC, and most BLBC are triple-negative breast cancers (TNBC), which are negative for estrogen receptor (ER), progesterone receptor (PR), and human epidermal growth factor receptor 2 (HER-2). In comparison to other types of BC, TNBC is more prevalent in younger women and has a more unfavourable prognosis due to its highly invasive, metastatic ability and high recurrence rate^4^, ^5^. Early diagnosis and treatment are keys to improving breast cancer survival rate, therefore, seeking and identifying specific biomarkers will help in targeted therapy, early diagnosis and prognosis of BC^6^-^8^.

As a member of the carcinoembryonic antigen (CEA) family, CEA related cell adhesion molecule-1 (CEACAM1) is a single-chain transmembrane glycoprotein belonging to the superfamily of immunoglobulin^9^. In addition to mainly mediating cell-to-cell adhesion, CEACAM1 has a variety of biological functions and participates in various physiological and pathological processes, such as cell proliferation, apoptosis, differentiation, lymphatic vessel neogenesis and angiogenesis^10^-^12^. Currently, studies have confirmed the strong relationship between CEACAM1 and the progression of tumor, and the CEACAM1 expression levels are generally dysregulated in most tumors^13^-^16^. It has been found that CEACAM1 expression is significantly up-regulated in cancers such as pancreatic^17^ and thyroid cancers^18^, which suggests that CEACAM1 may be a cancer-promoting molecule. However, other studies have also confirmed that CEACAM1 expression is significantly decreased in cancers such as hepatocellular carcinoma^19^, bladder cancer^20^, ^21^, kidney cancer^22^, etc., thereby indicating that CEACAM1 may function as a potential tumour suppressor. The reason for this contradiction may be related to the expression characteristics of CEACAM1 in different tumor tissues (cell membrane-type expression or cytoplasmic expression), but the exact reason is still not very clear. In any case, abnormal expression of CEACAM1 is of great significance in the diagnosis of tumors, the assessment of disease progression, and prognostic judgments^16^, ^23^.

According to different shearing modes, CEACAM1 has 12 isoform structures, of which CEACAM1-3L, 3S, 4L, and 4S are the major isoforms^24^. Different CEACAM1 isoforms play distinct regulatory roles in various tumors. It was found that both CEACAM1-4S and 4L overexpression could promote the invasive and metastatic ability of colon cancer cell line HT29^25^, ^26^, while the invasive and metastatic ability of hepatocellular carcinoma cell line HLF was impaired after overexpression of CEACAM1-4S and 4L^27^. Additionally, overexpression of both CEACAM1-4S and 4L were also reported to attenuate the invasive capability of gastric cancer cells NUGC3^28^. However, CEACAM1-4L, 3L and 4S were capable of promoting the ability of melanoma cells metastasis and invasion, while CEACAM1-3S significantly inhibited the invasion and metastasis of melanoma^29^. Previously, it has been reported that CEACAM1 expression was obviously deregulated in BC and might serve as a diagnostic biomarker in BC^30^, ^31^, but until now there are no reports on the relationship between CEACAM1 and prognosis of patients with BC.

Here the prognostic correlation between CEACAM1 and BLBC and its related potential mechanisms were firstly analysed by bioinformatics in the present study, and then we explored the regulatory roles of the major isoforms CEACAM1-3L and 4L on proliferation, invasion and metastasis of BC.

## Materials and methods

### Basal-like breast cancer cell lines

MDA-MB-231 (Procell, CL-0150, Wuhan, China), Hs-578T (Procell, CL-0114, Wuhan, China) and normal mammary epithelial cells MCF-10A (Procell, CL-0525, Wuhan, China) were all obtained from Pricella Biotechnology Co., Ltd.(Wuhan, China). Dulbecco's modified eagle's medium (DMEM) (Vivacell, C3113, Shanghai, China) were employed to cultivate MDA-MB-231 and Hs-578T cells. MCF-10A was incubated using specific epithelial culture medium (Procell, CL-0525, Wuhan, China). All cell culture media contained 10% fetal bovine serum (FBS) (Vivacell, C04001-500, Shanghai, China). Cells were all cultured in a humidified environment at 37 ℃, 5% CO_2_.

### Data acquisition

In the present study, gene expression data about CEACAM1 in breast cancer was sourced from the Cancer Genome Atlas (TCGA) database, but was downloaded directly from the database of UCSC Cancer Browser (https://xenabrowser.net/) and cbioportal (http://www.cbioportal.org/). The corresponding clinical information on subtypes, prognosis, and other information about BC samples were also downloaded from UCSC Cancer Browse.

### Survival analysis and Cox regression analysis

To investigate the association between CEACAM1 gene expression and survival of breast cancer patients with different subtypes, we collected data of BC patients from the TCGA database. For BC patients who were lost follow-up before death, the time of last follow-up was usually calculated as the time of death. Finally, 927 BC patients in the TCGA database were included in this study. Depending on the median level of CEACAM1 expression as the threshold, all subjects were divided into CEACAM1 high expression group and low expression group. The research used Kaplan-Meier survival analysis and compared the differences of the survival between two groups using the log-rank test.

To further appraise the potential of CEACAM1 as an independent prognostic factor for BLBC, cox regression analysis was carried out on the CEACAM1 data in the database of TCGA-BLBC, and hazard ratios (HRs) and 95% confidence intervals (CI) were calculated. Furthermore, alignment diagram was created to predict one-, three-, and five-year survival probabilities for BLBC patients. A total score was calculated based on the score corresponding to each factor, and the possibility of survival in BLBC was predicted by the total score.

### Gene Set Enrichment Analysis (GSEA)

The software of GSEA (http://www.broadinstitute.org/gsea) was used for the purpose of analysing signaling pathways associated with high and low CEACAM1 expression. CEACAM1 gene expression data of BLBC were analysed through the genome database (c2.cp.kegg.v7.4.symbols.gmt) in the software of GSEA. Results such as normalized enrichment scores (NES), FDR corrected pathways and p-values were acquired through the GSEA software. In general, the set of pathway genes with |NES| > 1 and FDR q-values < 0.05 were regarded as statistically significant.

### Differentially expressed genes (DEG)

The "limma" package in R software was utilized to examine DEG associated with high or low CEACAM1 expression. According to the Benjamini-Hochberg method, |log_2_(foldchange)| > 1 and p < 0.05 were chosen as the threshold for screening significantly DEG.

### Analysis of protein interaction networks

The online tools (https:/string-db.org/ and http://genemania.org) were used to screen genes whose corresponding proteins interact with CEACAM1.

### The pathway analysis of Kyoto Encyclopedia of Gene and Genome (KEGG)

Based on the CEACAM1-related DEG, KEGG pathway analysis were conducted using the package "clusterProfiler" in the software of R. p < 0.05 was deemed to be significantly enriched term. We used the ggplot2 package for R to present the top-ranked pathways in terms of the number of enriched mRNAs in a bubble plot.

### RT-qPCR

Cells were collected and RNA was extracted, and cDNA was synthesised using a reverse transcription kit (YEASEN, 11121ES60, Shanghai, China), followed by RT-qPCR using SYBR Green qPCR reagent (Biosharp, BL698A, Beijing, China). Moreover, RT-PCR for this study was performed using primers specific for human CEACAM1-4L (NM001712) were: F: 5'-AAACCAGAGTCTCCCGTCCT-3'; R: 5'-TTGTGCTCTCTGTGAGATCACGC-3'), and specific primers for human CEACAM1-3L (X14831) were: F: 5'-TCACTGATAATATGCTCTACCACAAGA-3'; R: 5'-TTGTGCTCTCTGTGAGATCACGC-3'. The primer for human GAPDH were: F: 5'-GCCATCACGTATCGTGGAAGG-3', R: 5'-GCCATCACGCCACAGTTTC-3'.

### Cell transfection

The empty vector of plasmids (Miaoling Biology, P40122, Wuhan, China) served as a control. Plasmids contained CEACAM1-3L and CEACAM1-4L genes were constructed. BLBC cells (MDA-MB-231 or Hs-578T) were seeded into 6-well plates (3 × 10^5^ cells/well) and 1.25μg of plasmid DNA was transfected using lipofectamine2000 reagent (Invitrogen, 11668-019, Carlsbad, USA). After 6 h of transfection, cells were rinsed and continued to be cultured in complete growth medium for 48 h, and then lysed for PCR analysis or other experiments.

### Cell counting kit-8 (CCK-8)

The suspension of BLBC cells (100 μL/well, 4500 cells/well) were seeded into 96-well plates and 5 μg of plasmid DNA was transfect using lipofectamine 2000. After culturing the cells for 48 h, 10 μL solution reagent of CCK-8 (Biosharp, BS350C, Beijing, China) was pipetted into each well. 2 h incubation later, the absorbance was detected at 450 nm and the change ratio in cellular activity was calculated.

### Analysis of apoptosis by flow cytometry

BLBC cells suspension in 6-well plate (100 μL/well, 10^5^~10^6^ cells/well) were pre-cultured for 24h, transfected 5 μg of plasmid DNA and continued to culture for 48h. The cells were then collected by trypsin digestion without EDTA, and washed twice with PBS and added 5 uL of Annexin V and 5 uL of Propidium Iodide (PI) (Biosharp, BL107B, Beijing, China). After reaction 15min at room temperature avoiding light, the proportion of apoptotic cells were analysed by flow cytometry.

### Transwell invasion assay

After overexpression transfection, MDA-MB-231 or Hs-578T cells (1000 per chamber) were seeded into a transwell chamber (LABSELECT, 14361, Shanghai, China) of a 24-well plate and incubated for 48 h. The cancer cells were fixed in the transwell chamber by methanol and stained with 0.1% crystal violet. Finally, the cancer cells in the lower layer of the chamber were photographed after rinsing twice with PBS.

### Wound-healing assay

MDA-MB-231 or Hs-578T cells in 6-well plates (1x10^5^ cells/well) were inoculated and cultured to achieve 100% fusion. The cultures were then scraped to form a wound line. After 24h, the wound area was observed with a microscope (magnification ×200; COIC microscope, Chongqing Shiguang, China) and the rate of cell migration was calculated.

### Statistical analysis

The statistical software of SPSS 20.0 (IBM SPSS, USA) and R software were used to analyse data of this study. Normally distributed data were described as mean ± standard deviation, and differences between groups were analysed by Student's t-test or one-way ANOVA. Kaplan-Meier survival analysis, cox regression analysis, alignment diagram, DEG analysis, KEGG, and Vann diagram were carried out by the software of R. p < 0.05 was deemed to be statistically significant.

## Results

### Data preprocessing and survival analysis

The mRNA expression data for breast cancer in TCGA downloaded from the UCSC Cancer Browser was log_2_(FPKM + 1), which was subsequently converted to log_2_(TPM + 1). The time frame of the 927 breast cancer samples sourced from the TCGA database ranged from May 2001 to October 2012. Based on molecular classification of the tumors, all samples included 490 Lum A subtypes (52.86%), 192 Lum B subtypes (20.71%), 77 Her2 subtypes (8.31%), and 168 Basal subtypes (18.12%) (Table 1). In 168 BLBC samples of our study, 59 were younger than 50 years and 109 were older than or equal to 50 years (Table 1). In addition, TNM stage in BLBC and the groups according to survival analysis parameters were also expressed as categorical variables (Table 1). However, there was a missing number of cases in the BLBC subgroup due to incomplete clinical data for some patients (Table 1).

### CEACAM1 expression levels in different subgroups of BLBC according to TCGA data

Depending on the data of TCGA-BLBC, we compared the levels of CEACAM1 mRNA in various clinical subgroups of BLBC and noticed that CEACAM1 expression were down-regulated in the subgroups with poorer prognosis (Table 2). Furthermore, in the four clinical variables including stage, OS, DSS, and PFS, the levels of CEACAM1 expression were significantly decreased in the poorer prognostic subgroup by comparison to the better prognostic subgroup (p = 0.0428, 0.0094, 0.0176, 0.0105, respectively) (Table 2 and Suppl. Figure S1). However, as some patients' clinical data were incomplete, this may have affected the comparison of CEACAM1 expression in subgroups of BLBC.

**Figure 1:**
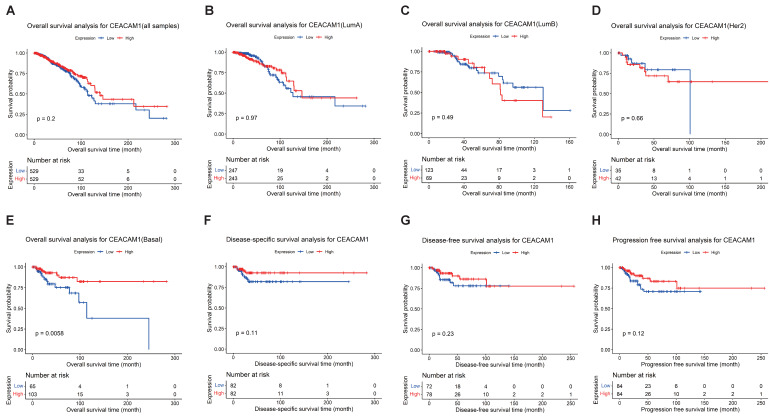
Survival analysis of CEACAM1 in BC on the basis of TCGA data. Overall survival analysis of CEACAM1 in BC and its subgroups (A-E), and DSS, DFS, and PFS analysis of CEACAM1 in BLBC (F-H).

We performed survival analyses of CEACAM1 expression in all breast cancer samples and observed no correlation between the levels of CEACAM1 expression and overall survival (OS) (Figure 1A-D). The survival analysis of CEACAM1 was also explored in different subtypes of BC patients, and we found that CEACAM1 was significantly correlated with OS in BLBC patients, and high CEACAM1 expression predicted a better prognosis (p = 0.0056)(Figure 1E). In addition, by examining the relationship between CEACAM1 expression and disease-specific survival (DSS), disease-free survival (DFS) and progression-free survival (PFS), it was also suggested that patients with high CEACAM1 expression had a better prognosis, however, the difference of statistics was not significant (p > 0.05)(Figure 1F-H).

### Regression analysis suggests that CEACAM1 may be an prognostic biomarker in BLBC

**Figure 2:**
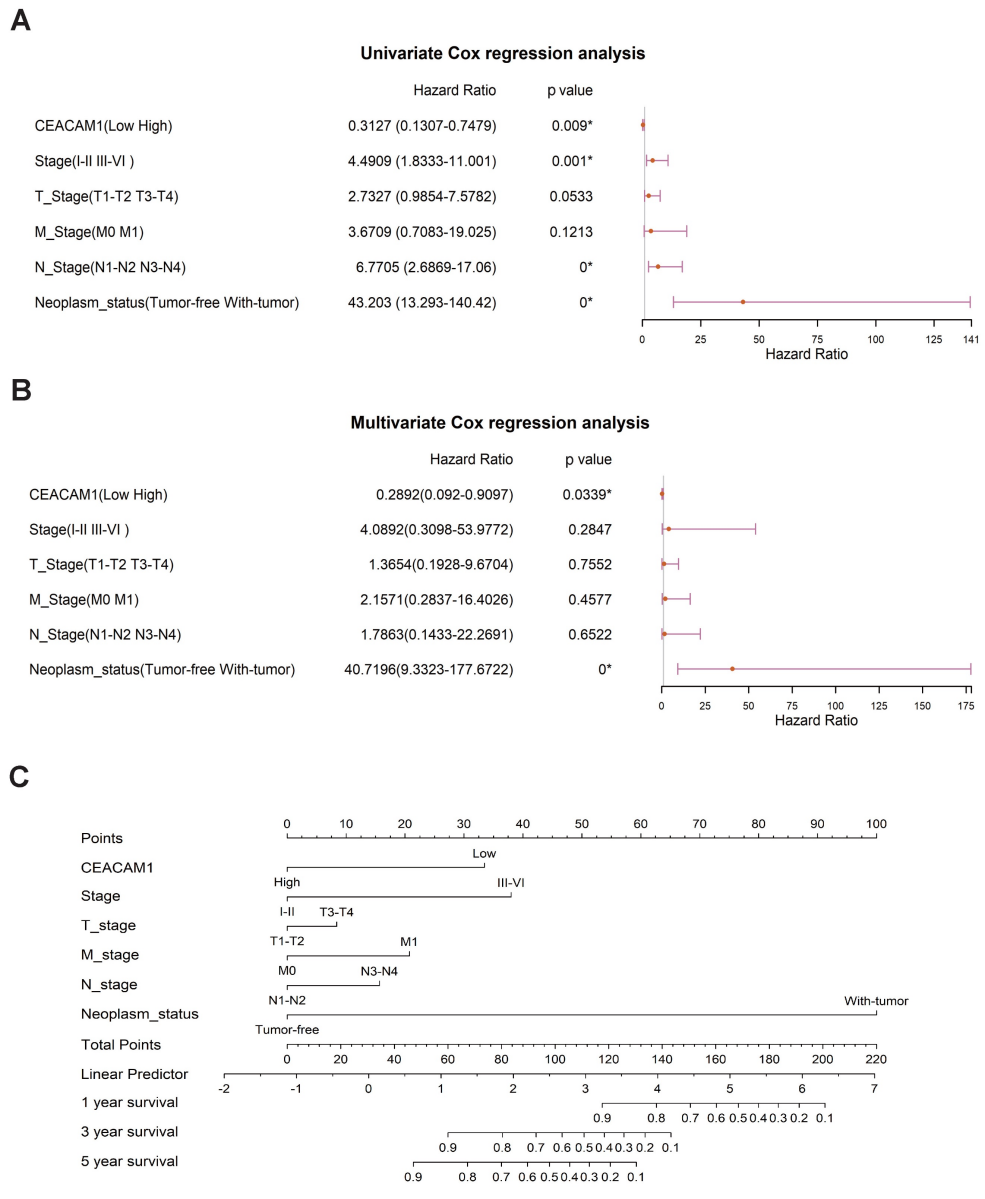
Cox regression analysis of CEACAM1 as a prognostic marker for BLBC. Forest plots of cox regression analysis displayed the association between CEACAM1 and clinicopathological parameters (stage, T-stage, N-stage, M-stage, tumor absence or presence) in patients with BLBC (A-B). Alignment diagram of the relationship between CEACAM1 and prognosis of BLBC patients (C).

Significant correlations between CEACAM1 expression and BLBC stage (I-II vs III-IV), N-stage (N1-N2 vs N3-N4), and tumor status (tumor absence vs presence) (p = 0.001, 0.000, < 0.0001, respectively) (Figure 2A) were obtained by univariate cox regression analysis. Multivariate cox regression analysis also showed a significant relationship between CEACAM1 expression and the status of tumor presence or absence in BLBC (p < 0.0001) (Figure 2B). Furthermore, we investigated the relationship between CEACAM1 expression and the 1, 3, 5-year survival of BLBC patients by plotting an alignment diagram, and the results showed a higher probability of longer survival in BLBC patients with high CEACAM1 expression (Figure 2C). The aforementioned results collectively indicate that CEACAM1 may be a potential prognostic biomarker in BLBC.

### GSEA analysis of CEACAM1-related signaling pathways

**Figure 3:**
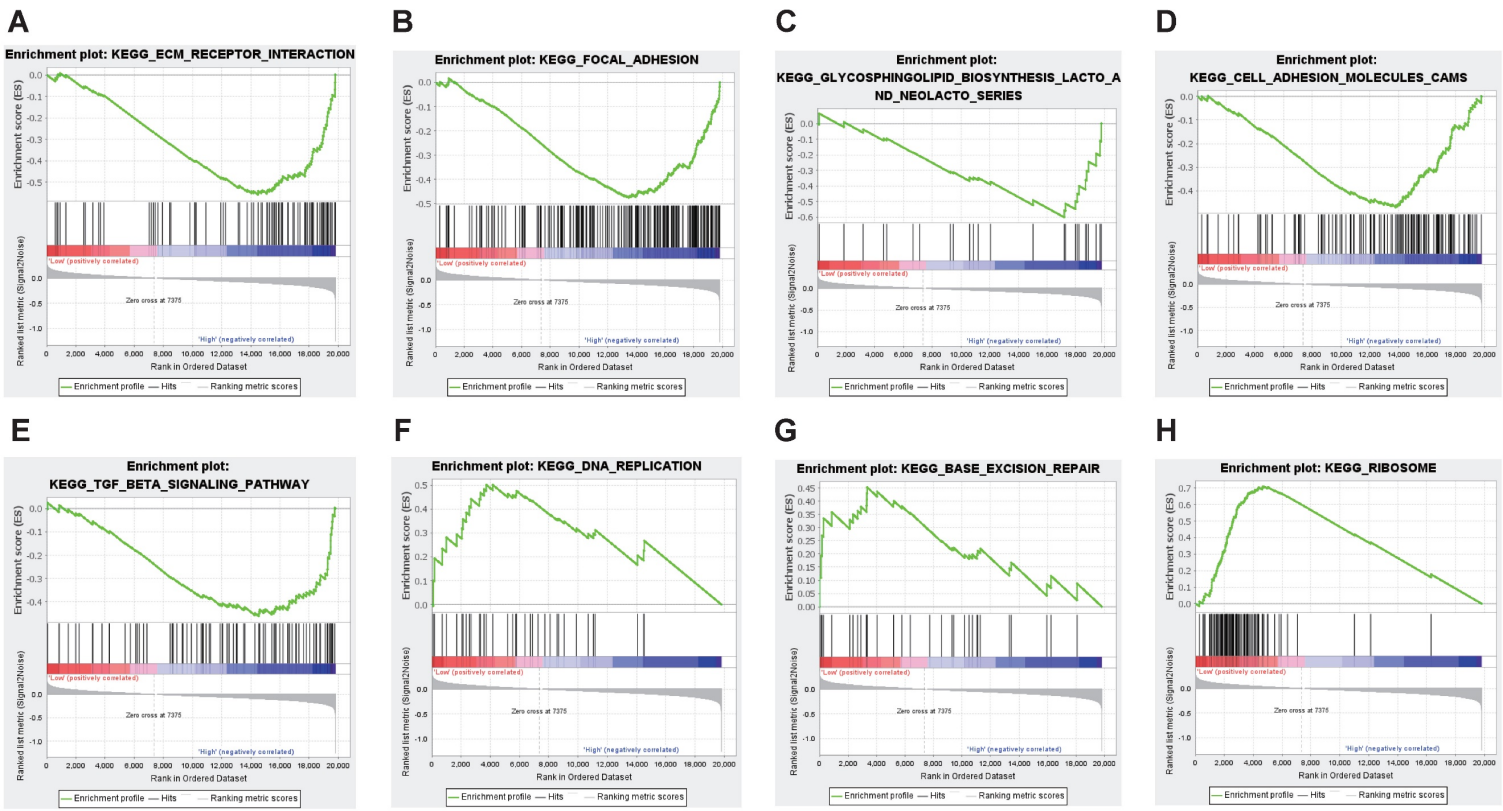
GSEA analysis of potential signaling pathways associated with CEACAM1. CEACAM1 was negatively related to pathways such as ECM receptor (A), focal adhesion (B), glycosphingolipid biosynthesis lacto and neolacto series(C), cell adhesion molecules CAMS (D), TGF-beta signaling (E). CEACAM1 was positively correlated with signaling pathways such as ribosome (F), DNA replication (G), base excision repair signaling pathways (H).

The discrepancy of prognosis between BLBC patients with high and low CEACAM1 expression may be related to some important signaling pathways. Therefore, we conducted GSEA analysis and depending on the set threshold, we obtained a total of 10 pathways enriched in CEACAM1 low expression set and 5 pathways enriched in CEACAM1 high expression set. These findings suggested that CEACAM1 was negatively related to signal pathways such as focal adhesion, extracellular matrix (ECM) receptor interaction, and cell adhesion, etc., while CEACAM1 was positively correlated with signaling pathways such as ribosome, DNA replication, base excision repair signaling pathways (Table 2 and Figure 3).

**Figure 4:**
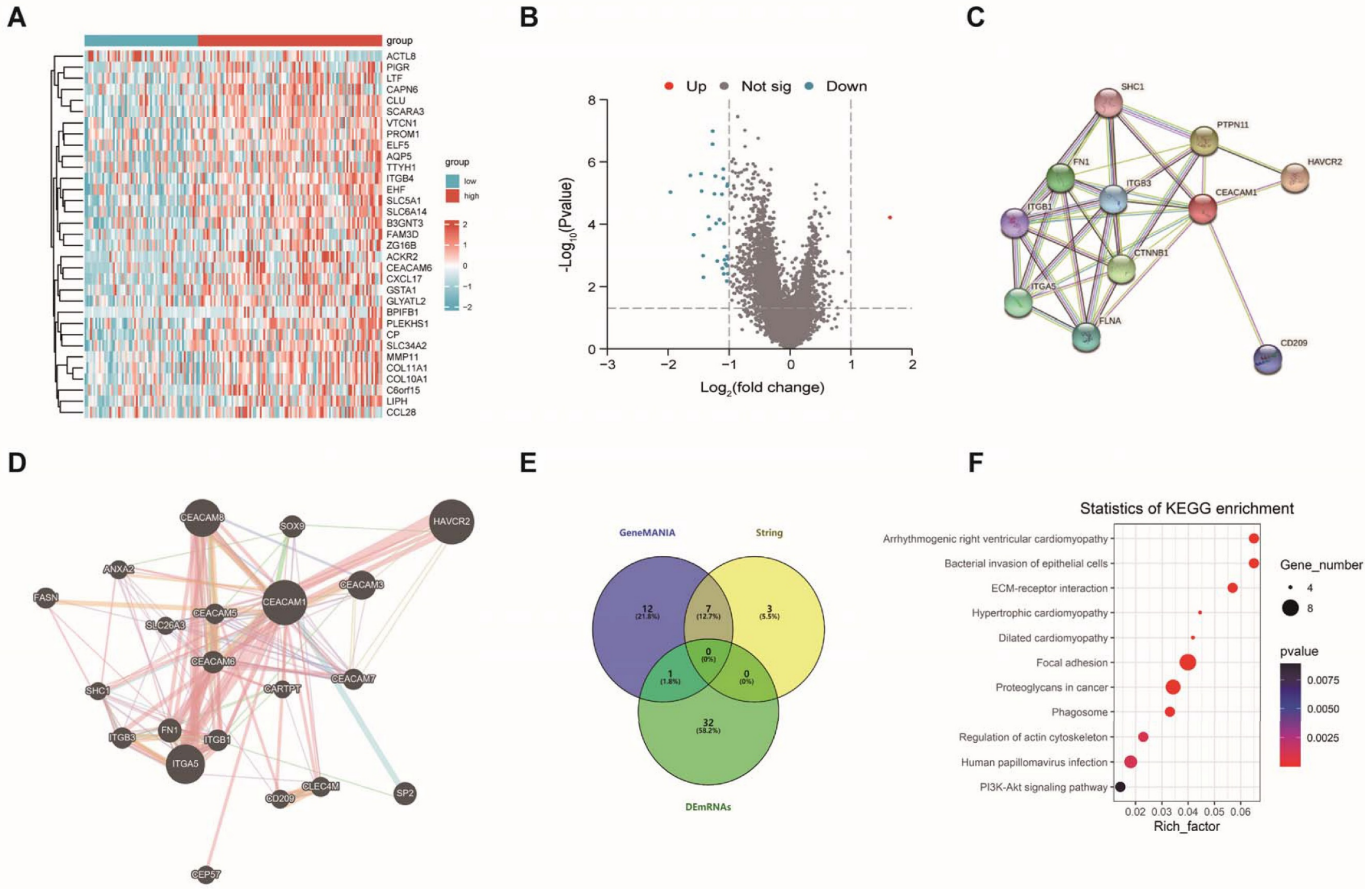
CEACAM1-related genes and its KEGG analysis. DEG between BLBC with high and low CEACAM1 expression (A-B). Genes that interacted with CEACAM1 were obtained by String and GeneMANIA (C-D). DEG and related genes obtained from String and GeneMANIA database were performed to produce a Vann diagram to find 55 CEACAM1-related genes (E). KEGG pathway analysis of signaling pathways with CEACAM1-related genes (F).

To further explore the function of CEACAM1, we firstly obtained 33 significantly differentially expressed genes that interacted with CEACAM1 in BLBC (Figure 4A-B). Among these genes, only one demonstrated an increase in expression, while 32 exhibited a decrease. And 10 and 20 reciprocal genes were also obtained by String and GeneMANIA, respectively (Figure 4C-D). These CEACAM1-related genes (Totally 55 genes, Figure 4E) were subjected to KEGG pathway analysis, and 23 significantly enriched pathways were found, and we also found that CEACAM1 was correlated with signaling pathways, such as focal adhesion, ECM-receptor interaction, etc (Figure 4F).

### CEACAM1 regulates proliferation and apoptosis in BLBC cells

**Figure 5:**
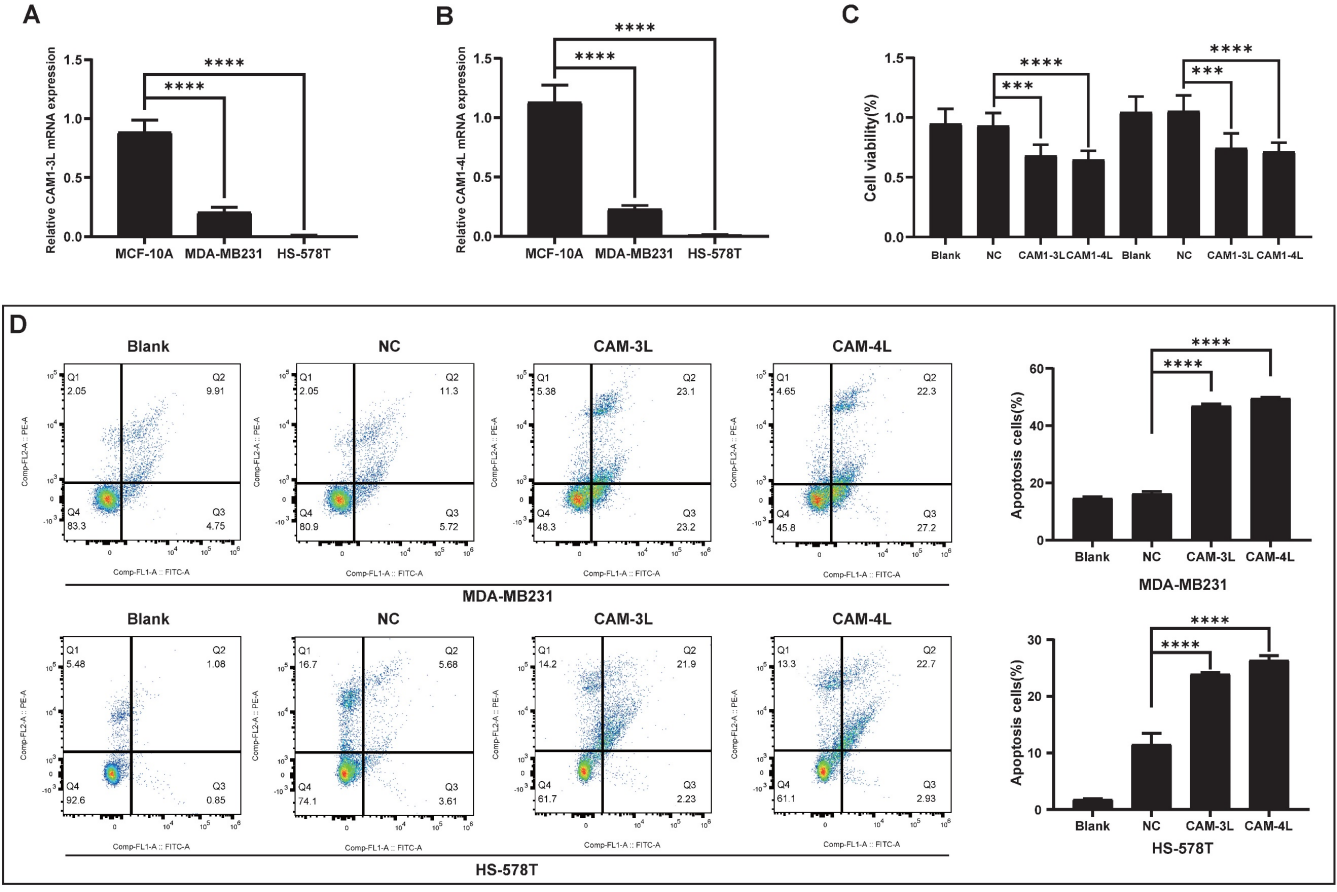
CEACAM1 expression in BLBC cells and its regulation of cell viability. mRNA expression of CEACAM1-3L and 4L in MCF-10A, MDA-MB-231, and Hs-578T (A-B). The proliferative activity of BLBC cells overexpressed CEACAM1-3L and 4L was measured by CCK-8 assays (C). Flow cytometric analysis of apoptosis in BLBC cells after overexpression of CEACAM1-3L and 4L (D). *** p < 0.001, *** p < 0.0001.

The relative mRNA expression of CEACAM1-3L and 4L in BLBC cells (MDA-MB-231 and Hs-578T) was obviously lower than that in normal cells (MCF-10A) by PCR experiments (Figure 5A-B). Based on the bioinformatics analysis, CEACAM1 was found to have the potential to regulate apoptosis in breast cancer, and overexpression of CEACAM1-3L and 4L in BLBC cells was identified to significantly suppress the proliferative activity of cancer cells by CCK-8 assay (Suppl. Figure S2 and Figure 5C). In addition, flow cytometric analysis of BLBC cells apoptosis after overexpression of CEACAM1-3L and 4L also showed that CEACAM1 remarkably promotes apoptosis of cancer cells (Figure 5D).

### CEACAM1 regulates invasion and migration of basal-like breast cancer cells

**Figure 6:**
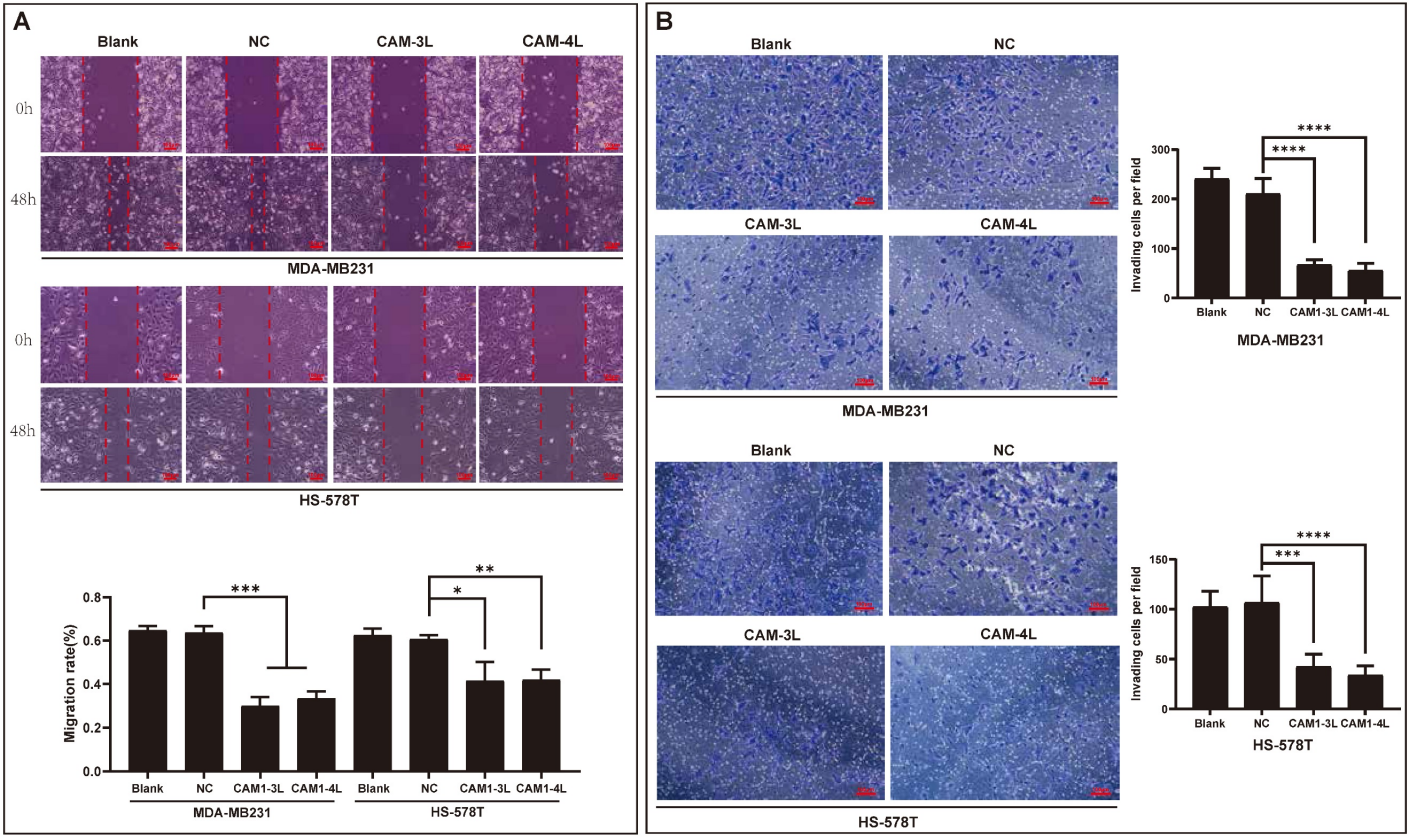
CEACAM1 inhibits BLBC cells migratory and invasive capacity. The migration rate (A) and number of invading cells (B) of MDA-MB-231 and Hs-578T overexpressing CEACAM1-3L and 4L were assessed by the experiments of wound-healing and transwell. *p < 0.05, ** p < 0.01, *** p < 0.001, *** p < 0.0001.

Since bioinformatics analysis suggested that CEACAM1 was significantly negatively related with prognosis of BLBC patients, and the main reason affecting the prognosis of BC patients might be invasion and metastasis. In the current study, the findings through wound-healing experiment also demonstrated that the migration rates of BC cells (MDA-MB-231 and Hs-578T) overexpressing CEACAM1-3L and 4L were significantly less than that in the groups of blank and negative control (NC) (Figure 6A). Moreover, in the transwell experiment, we similarly found that the number of invasive cancer cells in BC cells overexpressing CEACAM1-3L and 4L was also significantly less than that in the blank and NC groups (Figure 6B).

## Discussion

As one of the most common malignancies in female, the prognosis of breast cancer, especially BLBC, remains disappointing^1^, ^3^. Currently, breast cancers are often classified into four major subtypes based on molecular typing: Luminal A and Luminal B, basal-like, and HER2 overexpression. In particular, approximately 75% of BLBCs belong to TNBC, and the lack of ER, PR, and HER receptors limits the use of endocrine and targeted therapies. The prognosis of BLBC is usually related to tumor volume, grade and early recurrence. In addition, BLBC is highly invasive and metastatic, usually spreading to organs such as the brain, bone, and lung, which in turn leads to an unfavourable prognosis. Therefore, development of new biomarkers to elucidate the determinants of invasive and metastatic disease and thus improve the prognosis of this disease is imminent.

As an important molecule mediating cell-cell adhesion, CEACAM1 is also able to regulate cell proliferation, apoptosis, and lymphatic and vascular neogenesis. However, the expression of CEACAM1 in different tumors is contradictory. CEACAM1 is significantly up-regulated in gastric^32^, pancreatic^17^, and thyroid cancers^18^, but decreased in hepatocellular^19^, bladder^21^, and renal cancers^22^, for which the reasons were not completely clarified. Our study found that CEACAM1 expression was reduced in BLBC cells (MDA-MB-231 and Hs-578T) compared to normal breast cells (MCF-10A). Despite the paradoxical trends of CEACAM1 expression in different tumors, CEACAM1 has been verified to play a pivotal role during tumor development. Therefore, the biofunction and clinical values of CEACAM1 in BC remain to be further ascertained. In the present study, we conducted an investigation of the expression level, prognostic significance, and biological function of CEACAM1 in BLBC for the first time.

There is a close relationship between abnormal alterations in CEACAM1 expression and tumor progression and prognosis. Previously it was found that the survival of CEACAM1-negative lung adenocarcinoma patients was significantly longer than that of CEACAM1-positive patients^33^. Since there was a significant relationship observed between CEACAM1 and microvessel density (MVD), patients with esophageal squamous carcinoma with high CEACAM1 expression had poorer survival^34^. However, by immunohistochemical analysis of 235 gastric cancer patients, the results revealed that patients with high CEACAM1 expression had significantly longer survival compared with those with low CEACAM1 expression^28^. Furthermore, immunohistochemical analysis of tumor tissue microarrays from 17,747 patients with prostate cancer confirmed that absence of CEACAM1 expression predicted a poor prognosis in prostate cancer^35^. In the present study, by analysing the bioinformatics data, our findings demonstrated that CEACAM1 expression levels were related with survival of BLBC patients, and patients with high CEACAM1 expression exhibited a favourable prognosis. Furthermore, the expression of CEACAM1 was markedly lower in the subgroup with poorer prognosis than in the subgroup with better prognosis for four clinical parameters: stage, OS, DSS, and PFS. Subsequently, cox regression analysis demonstrated a significant relationship between CEACAM1 and the stage of BLBC, which further suggests that CEACAM1 is an potential prognostic marker for BLBC patients.

To further reveal the relationship between CEACAM1 and BLBC prognosis, the biofunctions of CEACAM1 in the pathogenesis of BLBC need to be ascertained. We firstly performed GSEA analysis, which revealed that CEACAM1 was negatively correlated with focal adhesion, ECM receptor interaction, and cell adhesion, but positively correlated with Ribosome, DNA replication, Base excision repair and other pathways. Then, KEGG pathway analysis was performed on DEG between the CEACAM1 low and high expression patients. These differentially expressed genes identified in our study were engaged in the development of breast cancer, including focal adhesion, the interaction of ECM receptor, and PI3K-Akt pathway. The relatively consistent findings were confirmed in oral cancer, where CEACAM1 was associated with these signaling pathways, and low expression of CEACAM1 in oral cancer led to worse prognosis^36^. The modulation between ECM and cells through focal adhesion, ECM receptors and actin cytoskeleton influences the morphology, adhesion and migration status of cells, which is important for tumor invasion and metastasis^37^-^39^. In addition, PI3K-Akt pathway is also involved in breast cancer proliferation and invasion^40^. Thus, CEACAM1 may modulate these pathways to influence BLBC occurrence and metastasis. Recently, it has been confirmed that decreasing CEACAM1 expression in liver cancer cells (Mahlavu and SK-Hep-1) may inhibit invasion and migration of tumor cells^41^. To further validate the suppressive properties of the CEACAM1 molecule on BC cells in *in vitro* experiments, we chose the main protein isoforms of CEACAM1-3L and 4L for the study. However, our results revealed that overexpression of CEACAM1-3L and 4L not only induced apoptosis and inhibited the viability of BLBC cells, but also inhibited the invasive and metastatic potential of cancer cells. Therefore, CEACAM1 may contribute to the OS of BLBC patients by inhibiting the proliferation and metastasis of cancer cells.

In our study, we sought to ascertain the correlation between CEACAM1 and prognostic value in BLBC, as well as gained preliminary insight into the biological functions of CEACAM1-3L and 4L in BLBC. However, it is undeniable that there are still potential limitations of this study that deserve further consideration. First, although bioinformatics analysis indicated that low expression of CEACAM1 predicted an unfavorable prognosis for BLBC patients, a large number of clinical trials are required to be conducted to confirm our results. Second, we predicted signaling pathways associated with CEACAM1 based on online databases, but absence of relevant experimental evidence.

## Conclusion

This study revealed that low CEACAM1 expression was strongly correlated with poor prognosis in BLBC by bioinformatics. The outcomes of GSEA and KEGG pathway analysis also suggested that CEACAM1 was engaged in regulating the proliferation and metastasis of BLBC. Moreover, we demonstrated the main isoforms of CEACAM1 suppressed the proliferation, invasion and metastasis of BLBC by cytological experiments. Therefore, CEACAM1 may improve patients' OS by inhibiting the proliferative ability and metastasis of BLBC. In the future, more attention should be paid to CEACAM1 and its different isoforms in the study of BLBC, the related molecular mechanisms should be further explored through more experiments, and the clinical significance of CEACAM1-3L or CEACAM1-4L in BLBC patients will be investigated.

## Supplementary Material

Supplementary figures.

## Figures and Tables

**Table 1:** Clinicopathological characteristics of the BC patients from TCGA database.

Group	Variable	Number	(%)
Subtypes	LumA	490	52.86
	LumB	192	20.71
	Her2	77	8.31
	Basal	168	18.12
Basal group			
Age	< 50	59	35.12
	≥ 50	109	64.88
Stage	I	21	12.65
	II-VI	145	87.35
T classification	T1	31	18.56
	T2-T4	136	81.44
M classification	M0	147	98.00
	M1	3	2.00
N classification	N1-N2	151	89.88
	N3	17	10.12
Tumor status	Tumor free	136	91.89
	With tumor	12	8.11
OS times (months)	Alive	146	86.90
	Dead	22	13.10
DSS times (months)	Alive or dead tumor free	150	91.46
	Dead with tumor	14	8.54
PFS times (months)	Censored	143	85.12
	Progression	25	14.88
DFS times (months)	DiseaseFree	133	88.67
	Recurred/Progressed	17	10.12

**Table 2:** CEACAM1 expression levels in different clinical subgroups of BLBC patients from TCGA database.

Variable	Group	Number	Mean ± SD	p
Age	< 50	59	4.6266 ± 1.4976	0.2984
	≥ 50	109	4.3490 ± 1.7075	
Stage	Ⅰ	21	5.1329 ± 1.5789	0.0428*
	Ⅱ-Ⅳ	145	4.3543 ± 1.6296	
T classification	T1	31	4.4785 ± 1.6046	0.4923
	T2-T4	136	4.2071 ± 1.9137	
M classification	M0	147	4.5544 ± 1.6204	0.6056
	M1	3	4.0652 ± 0.9752	
N classification	N1-N2	151	4.5046 ± 1.6687	0.1734
	N3	17	3.9300 ± 1.2734	
Tumor status	Tumor free	136	4.4002 ± 1.6343	0.2448
	With tumor	12	3.8292 ± 1.3415	
OS	Alive	146	4.5739 ± 1.6085	0.0094*
	Dead	22	3.6011 ± 1.6130	
DSS	Alive or dead tumor free	150	4.5200 ± 1.6224	0.0176*
	Dead with tumor	14	3.4400 ± 1.3555	
PFS	Censored	143	4.5817 ± 1.6318	0.0105*
	Progression	25	3.6732 ± 1.4796	
DFS	Disease Free	133	4.5955 ± 1.6447	0.0617
	Recurred/Progressed	17	3.8061 ± 1.3804	

## Abbreviations

BC: breast cancer
BLBC: basal-like breast cancer
TNBC: triple-negative breast cancer
ER: estrogen receptor
PR: progesterone receptor
HER-2: human epidermal growth factor receptor 2
CEA: carcinoembryonic antigen
CEACAM1: CEA related cell adhesion molecule-1
TCGA: The Cancer Genome Atlas
GSEA: Gene Enrichment Analysis
KEGG: Kyoto Encyclopedia of Gene and Genome
HRs: hazard ratios
CI: confidence intervals
OS: overall survival
ECM: extracellular matrix
DSS: Disease-specific Survival
DFS: Disease-Free Survival
PFS: Progression-Free Survival
RT-qPCR: Real-time fluorescence quantitative polymerase chain reaction
CCK-8: Cell Counting Kit-8
