# Supplementary material for: Identifying CEACAM1 as a potential prognostic biomarker for basal-like breast cancer by bioinformatics analysis and in vitro experiments
Source: J Cancer. 2024 Oct 21;15(19):6468–78. doi: 10.7150/jca.101636 (PMC11540499; doi:10.7150/jca.101636)
Supplement: Supplementary file 1 — Supplementary figures. [file jcav15p6468s1.pdf]

Supplementary Figure 1

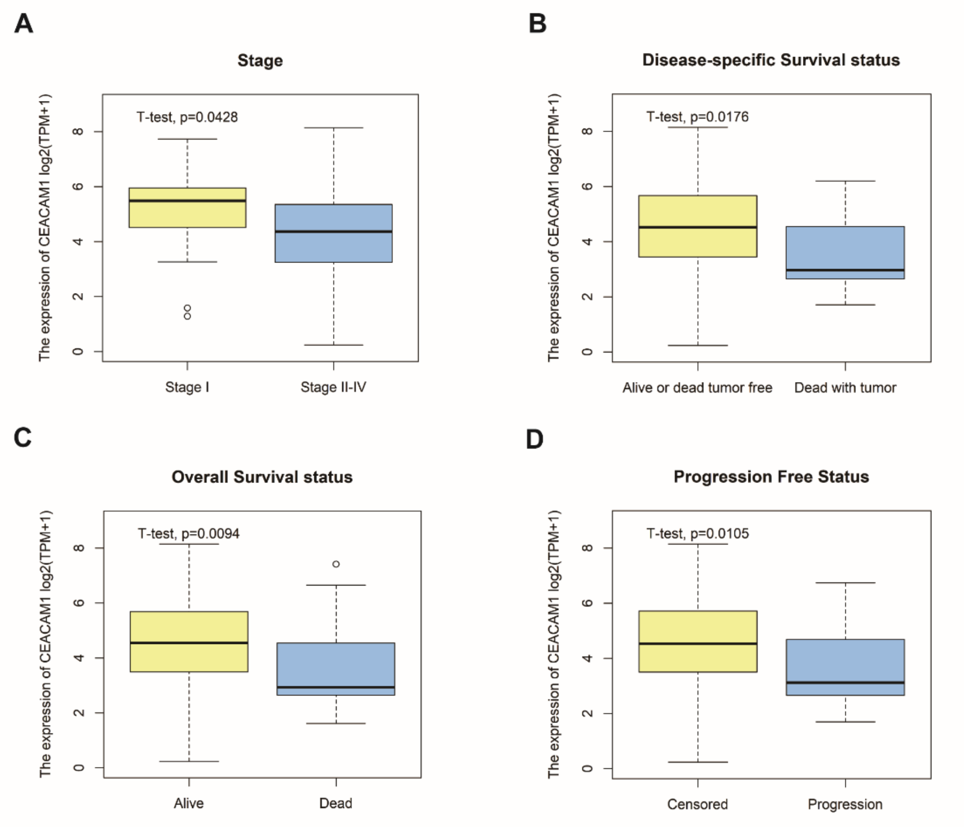

Supplementary Figure 1 Low CEACAM1 expression levels in the subgroup with poorer prognosis. The expression levels of CEACAM1 in the subgroups with poorer prognosis for the four clinical characteristics of Stage (A), DSS (B), OS(C), and PFS (D) were significantly lower than the subgroups with better prognosis.

Supplementary Figure 2

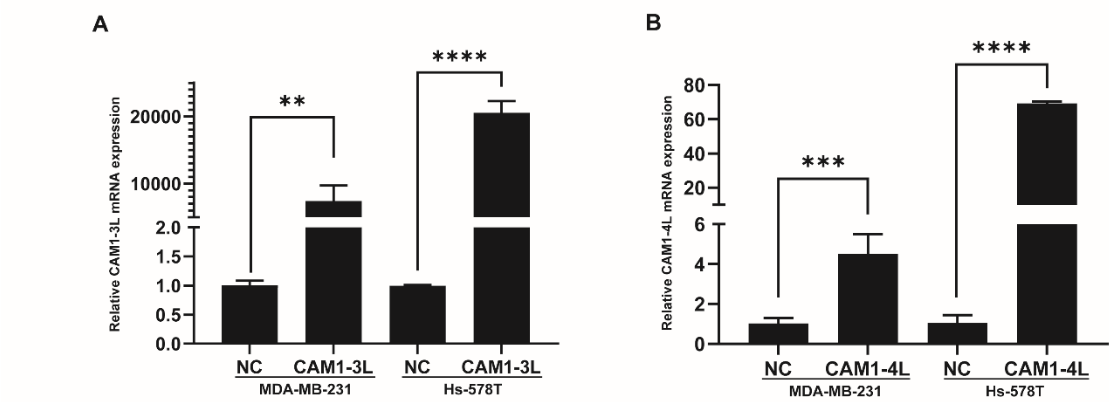

Supplementary Figure 2 Overexpressing CEACAM1 expression in BLBC cells. Expression of CEACAM1-3L (A) and CEACAM1-4L (B) mRNA in breast cancer cells (MDA-MB-231 and HS-578T) after overexpression. ** $p < 0.01$, *** $p < 0.001$, *** $p < 0.0001$.
